# The association of kidney function and cognitive decline in older patients at risk of cardiovascular disease: a longitudinal data analysis

**DOI:** 10.1186/s12882-020-01745-5

**Published:** 2020-03-05

**Authors:** Laurien E. Zijlstra, Stella Trompet, Simon P. Mooijaart, Marjolijn van Buren, Naveed Sattar, David J. Stott, J. Wouter Jukema

**Affiliations:** 1 Department of Cardiology, Leiden University Medical Center, Albinusdreef 2, 2333ZA, Leiden, The Netherlands; 2 Department of Gerontology and Geriatrics, Leiden University Medical Center, Albinusdreef 2, 2333ZA, Leiden, The Netherlands; 3 Department of Nephrology, Leiden University Medical Center, Albinusdreef 2, 2333ZA, Leiden, The Netherlands; 4 Department of Internal Medicine, HagaHospital, Els Borst-Eilersplein 275, 2545AA, The Hague, The Netherlands; 5 BHF Glasgow Cardiovascular Research Centre, Faculty of Medicine, Glasgow, G12 8TD UK; 6 Institute of Cardiovascular and Medical Sciences, College of Medical, Veterinary and Life Sciences, University of Glasgow, 126 University Place, G12 8TA, Glasgow, UK

**Keywords:** Cognitive function, Chronic kidney disease, Vascular disease

## Abstract

**Background:**

Chronic kidney disease (CKD) has been identified as a significant direct marker for cognitive decline, but controversy exists regarding the magnitude of the association of kidney function with cognitive decline across the different CKD stages. Therefore, the aim of this study was to investigate the association of kidney function with cognitive decline in older patients at high risk of cardiovascular disease, using data from the PROspective Study of Pravastatin in the Elderly at Risk (PROSPER).

**Methods:**

Data of 5796 patients of PROSPER were used. Strata were made according to clinical stages of CKD based on estimated glomerular filtration rate; < 30 ml/min/1.73m^2^ (stage 4), 30-45 ml/min/1.73m^2^ (stage 3b), 45-60 ml/min/1.73m^2^ (stage 3a) and ≥ 60 ml/min/1.73m^2^ (stage 1–2). Cognitive function and functional status was assessed at six different time points and means were compared at baseline and over time, adjusted for multiple prespecified variables. Stratified analyses for history of vascular disease were executed.

**Results:**

Mean age was 75.3 years and 48.3% participants were male. Mean follow-up was 3.2 years. For all cognitive function tests CKD stage 4 compared to the other stages had the worst outcome at baseline and a trend for faster cognitive decline over time. When comparing stage 4 versus stage 1–2 over time the estimates (95% CI) were 2.23 (0.60–3.85; *p* = 0.009) for the Stroop-Colour-Word test, − 0.33 (− 0.66–0.001; *p* = 0.051) for the Letter-Digit-Coding test, 0.08 (− 0.06–0.21; *p* = 0.275) for the Picture-Word-Learning test with immediate recall and − 0.07 (− 0.02–0.05; *p* = 0.509) for delayed recall. This association was most present in patients with a history of vascular disease. No differences were found in functional status.

**Conclusion:**

In older people with vascular burden, only severe kidney disease (CKD stage 4), but not mild to modest kidney disease (CKD stage 3a and b), seem to be associated with cognitive impairment at baseline and cognitive decline over time. The association of severe kidney failure with cognitive impairment and decline over time was more outspoken in patients with a history of vascular disease, possibly due to a higher probability of polyvascular damage, in both kidney and brain, in patients with proven cardiovascular disease.

## Background

Both chronic kidney disease (CKD) and cognitive impairment are increasingly prevalent with advancing age and partly share a common cause as the kidney and the brain share similar hemodynamic characteristics [1–3]. Both organs are low resistance end organs exposed to high-volume blood flow and therefore predisposed for vascular damage [4]. Next to aging, classical vascular risk factors like hypertension, diabetes and a history of cardiovascular diseases are associated with microvascular damage and small vessel disease in both the kidney and the brain [5–10].

Furthermore, CKD has been identified as a significant marker for cognitive impairment [11, 12]. Although the underlying pathophysiological mechanisms of cognitive dysfunction in CKD remain largely unknown, several candidate mechanisms have been suggested apart from cardiovascular risk factors, which are the same for kidney and brain. In addition, nephrogenic risk factors as uremic toxins, oxidative stress, anaemia, albuminuria and inflammation can lead to cognitive impairment [10, 13]. Also in end-stage kidney disease determinants related to dialysis, such as intradialytic hypotension or cerebral oedema can lead to cerebral hypoperfusion or neuronal damage [10, 12].

The prevalence and magnitude of the association of cognitive impairment across different CKD stages is still subject of debate. Whereas the relationship has been firmly established in patients with end-stage kidney disease, the association of mild to modest impaired kidney function with cognitive function remains questionable [14].

We hypothesized that with decreasing kidney function, cognitive function declines faster over time. Therefore, the aim of this study was to investigate the association of the different stages of CKD and cognitive decline and functional status in a high-risk population of older patients and furthermore, to investigate this association in patients with a history of vascular disease, or patients with only vascular risk factors, using data from the PROspective Study of Pravastatin in the Elderly at Risk (PROSPER).

## Methods

The study population comes from PROSPER, a double-blind, randomized, placebo-controlled trial, designed to investigate the relationship between statin treatment and the risk of cardiovascular and cerebrovascular events. In summary, 5804 older participants (70–82 years) were enrolled in Ireland, Scotland and The Netherlands. Patients were included if they had a history of, or an increased risk for vascular disease and a baseline cholesterol between 4.0–9.0 mmol/l. A history of vascular disease included stroke, transient ischemic attack, myocardial infarction, arterial surgery, or amputation for vascular disease less than 6 months before study entry. Increased risk for vascular disease included current smoking, hypertension, known diabetes mellitus or fasting blood glucose levels over 7 mmol/L. Mean follow-up was 3.2 years. Detailed description of this population, including all in- and exclusion criteria, has been published previously [15]. The study was approved by the institutional ethics review boards of centres of Cork University (Ireland), Glasgow University (Scotland) and Leiden University Medical Center (the Netherlands). Consent has been obtained from each patient or subject after full explanation of the purpose and nature of all procedures used.

### Kidney function

At baseline creatinine levels were measured. Individuals with baseline creatinine levels over 200 μmol/l were excluded. GFR was estimated using the Modification of Diet in Renal Disease equation: eGFR = 186 x Scr^(− 1.154)^ x age^(− 0.203)^ × 0.742 [if female], where Scr denotes serum creatinine level in mg/dl. It is assumed that all participants were of Northern European descent [16]. Statistical analysis of baseline characteristics was based on a comparison among subgroups of clinical stages of kidney failure based on eGFR, namely < 30 (CKD stage 4), 30–45 (CKD stage 3b), 45–60 (CKD stage 3a) and ≥ 60 ml/min/1.73m^2^ (CKD stage 1–2) [17].

### Cognitive function and functional status

Detailed description of the cognitive function and functional status measurements in PROSPER has been published previously [18, 19]. Measurement of cognitive function and functional status were prespecified endpoints. One of the exclusion criteria was a poor cognitive function at baseline, measured by the Mini-Mental State Examination (MMSE). We used the generally used cut-off point of 24 (of 30) points.

Outcome variables were derived from three other widely used neuropsychological performance tests in different cognitive domains and two functional status tests, as decline in functional status is largely driven by cognitive impairment. First, executive functioning was assessed using the Stroop Colour Word Test (Stroop) and the Letter-Digit Coding Test (LDT). Selective attention was assessed using Stroop, which consist of three parts, namely colour names, coloured patches and colour names printed in incongruously coloured ink. The time in seconds required to read the names or to identify colours is recorded [20]. We used an abbreviated version of the test with 40 elements [21]. Processing speed of general information was assessed using the LDT, which is a modification of the procedurally identical Symbol-Digits Modalities Test, which has an outcome variable of total number of correct entries completed in 60 s [22, 23]. Second, memory was assessed using the Picture-Word Learning Test. This verbal learning test is derived from the Groningen 15 Words Test. Outcomes are measured in three different trials and divided in recall (PLTi) and delayed recall (PLTd) after 20 min [24–26]. Functional status was assessed using two questionnaires, namely the Barthel Index and the Lawton Instrumental Activities of Daily Living Scale (IADL) [27, 28]. Barthel measures performance in basic activities of daily living and consists of 10 items. Barthel scores range from 0 to 20, with lower scores indicating more dependence. IADL evaluates more complex instrumental activities and includes 7 items. IADL scores range from 0 to 14, with again lower scores indicating more dependence. Cognitive function and functional status were tested at several time points, namely at baseline and after 9, 18 and 30 months, and at the end of the study.

### Statistical analysis

All categorical data are presented as numbers with percentages and were compared using the chi-square test. All continuous data are presented as mean ± standard deviation or median with interquartile range and were compared using an one-way ANOVA or Kruskal-Wallis test.

Means of cognitive function test scores and functional status scores at baseline were compared between the different CKD stages using a one-way ANOVA test. Furthermore, severe CKD stage 4 was compared to stage 1–2 (no CKD) using an independent t-test. For follow-up linear mixed models for repeated measurements were used, including the interim measures taken between the baseline and the final assessment. This last measurement varies between all participants between 36 and 48 months. Therefore all statistical analyses are performed with their individually varying time point, but we graphically display the results for the mean of these time points at 42 months. To preclude possible learning effects the pre-randomized measurement was discarded in all analyses. From the first PROSPER article about cognitive function, we know that all cognitive tests show a significant decline over time, confirming their adequate sensitivity to pick up deterioration of cognitive function in old age [19]. In the mixed model analyses CKD stages, time and CKD stage * time were included. Furthermore, to correct for confounders multiple prespecified fixed effects were included, namely sex, age, educational status, country, statin treatment, vascular confounders including history of vascular disease, hypertension, diabetes and current smoking, and other known confounders including objective measures as blood pressure, BMI, baseline lipids, haemoglobin, urea, NT-proBNP and troponin, all assessed as previously reported [15]. A log-transformation will be used for the variables with skewed distribution. Analyses will be repeated stratified for subjects with or without a history of vascular disease. The data were analysed using IBM SPSS Statistics version 23. *P*-values lower than 0.05 were considered statistically significant.

## Results

Of the 5804 randomised patients, baseline creatinine levels were available for 5796 participants (99.9%). Participants had a mean age of 75.3 years and 48.3% were male. Mean eGFR was 60.0 ± 14.6 ml/min/1.73m^2^. In the prespecified stages of CKD, based on eGFR, 19 subjects (0.33%) had a baseline eGFR of < 30 ml/min/1.73 m2 (CKD stage 4), 786 (13.6%) an eGFR between 30 and 45 ml/min/1.73 m2 (stage 3b), 2306 (39.8%) an eGFR between 45 and 60 ml/min/1.73 m2 (stage 3a), and 2685 (46.3%) an eGFR ≥60 ml/min/1.73m^2^ (stage 1–2).

Baseline characteristics are shown in Table 1, over strata of CKD stage and overall. Lower eGFR was significantly associated with older age, female sex, less years of education, more history of hypertension, vascular disease and medication use, and less history of diabetes and current smoking. Furthermore, lower eGFR was associated with an unfavorable lipid profile, higher levels of CRP, urea, NT-proBNP and troponin-T and lower levels of hemoglobin.

**Table 1:** Baseline Characteristics Split by Baseline CKD stages and Overall

	**Total** ***n*** **= 5796**	**CKD stages based on eGFR (ml/min/1.73m**^**2**^**)**				*P*-value*
		**Stage 4** ***n*** **= 19**	**Stage 3b** ***n*** **= 786**	**Stage 3a** ***n*** **= 2306**	**Stage 1 and 2** ***n*** **= 2685**	
Age (years)	75.3 ± 3.3	77.4 ± 3.1	76.8 ± 3.4	75.3 ± 3.3	74.9 ± 3.2	< 0.001
Male gender	2799 (48.3)	0	223 (28.4)	1029 (44.6)	1547 (57.6)	< 0.001
Education (years)	15.1 ± 2.0	15.1 ± 1.8	14.8 ± 1.6	15.0 ± 1.8	15.4 ± 2.3	< 0.001
History of hypertension	3585 (61.9)	18 (94.7)	568 (72.3)	1471 (63.8)	1528 (56.9)	< 0.001
History of diabetes	622 (10.7)	1 (5.3)	68 (8.7)	221 (9.6)	332 (12.4)	0.002
History of vascular disease	2561 (44.2)	7 (36.8)	400 (50.9)	1062 (46.1)	1092 (40.7)	< 0.001
History of stroke or TIA	647 (11.2)	1 (5.3)	86 (10.9)	271 (11.8)	289 (10.8)	0.584
Current smoker	1558 (26.9)	4 (21.1)	131 (16.7)	558 (24.2)	865 (32.2)	< 0.001
Number of medications	3.6 ± 2.3	5.2 ± 2.8	4.5 ± 2.3	3.7 ± 2.3	3.2 ± 2.2	< 0.001
SBP (mmHg)	154.7 ± 21.8	156.1 ± 27.3	154.8 ± 22.2	154.6 ± 21.5	154.6 ± 22.0	0.728
DBP (mmHg)	83.8 ± 11.4	82.5 ± 11.2	83.1 ± 10.7	83.6 ± 11.4	84.1 ± 11.6	< 0.001
BMI (kg/m^2^)	26.8 ± 4.2	26.6 ± 3.8	27.6 ± 4.4	26.8 ± 4.2	26.6 ± 4.1	< 0.001
LDL-C (mmol/l)	3.79 ± 0.80	4.06 ± 0.93	3.92 ± 0.81	3.85 ± 0.81	3.71 ± 0.78	< 0.001
HDL-C (mmol/l)	1.28 ± 0.35	1.29 ± 0.38	1.25 ± 0.34	1.28 ± 0.35	1.29 ± 0.35	< 0.001
Total cholesterol (mmol/l)	5.68 ± 0.91	6.02 ± 1.03	5.88 ± 0.94	5.74 ± 0.87	5.57 ± 0.87	< 0.001
Triglyceride (mmol/l)	1.54 ± 0.70	1.85 ± 0.74	1.76 ± 0.77	1.57 ± 0.69	1.46 ± 0.68	< 0.001
Glucose (mmol/l)	5.5 ± 1.4	5.13 ± 0.94	5.56 ± 1.40	5.50 ± 1.31	5.4 ± 1.6	< 0.001
CRP at 6 months (mg/l)	2.3 [1.1–4.5]	4.2 [2.1–10.1]	2.8 [1.4–5.7]	2.3 [1.1–4.6]	2.1 [1.0–4.1]	< 0.001
Urea (mg/dL)	6.3 ± 1.8	10.5 ± 1.8	7.9 ± 2.3	6.4 ± 1.6	5.8 ± 1.5	< 0.001
Hb (mmol/L)	8.7 ± 0.8	8.4 ± 0.7	8.4 ± 0.8	8.7 ± 0.8	8.8 ± 0.8	< 0.001
NT-proBNP at 6 months (ng/l)	148.7 [79.5–289.3]	417.0 [283.9–789.0]	230.2 [122.1–489.1]	151.5 [81.4–293.9]	127.6 [70.0–238.8]	< 0.001
Troponin at 6 months (μg/l)	0.010 ± 0.036	0.016 ± 0.010	0.013 ± 0.017	0.010 ± 0.042	0.010 ± 0.036	< 0.001

All values are presented as n (%), mean ± SD or median [IQR]. * *p*-values of categorical data were assessed using the chi-square test and *p*-values of the continuous data were assessed using an one-way ANOVA test or a Kruskal-Wallis test. Abbreviations: *BMI* Body mass index, *CKD* Chronic kidney disease, *CRP* C-reactive protein, *DBP* Diastolic blood pressure, *eGFR* Estimated glomerular filtration rate, *Hb* Haemoglobin, *HDL* High-density lipoprotein, *LDL* Low-density lipoprotein, *NT-proBNP* N-terminal pro b-type natriuretic peptide, *SBP* Systolic blood pressure, *SD* Standard deviation, *TIA* Transient ischemic attack

A higher score for Stroop or a lower score for the other five tests indicate a worse cognitive function or functional status. Non-adjusted baseline cognition and functional status scores are shown in Table 2, over strata of CKD stage and overall. The participants with the most impaired kidney function (CKD stage 4) had the worst cognitive function and functional status in all domains at baseline. When comparing the CKD stage 4 versus stage 1–2 mean scores (± SE) were 74.2 ± 6.7 vs 69.3 ± 0.6 for Stroop (*p* = 0.514), 21.9 ± 1.2 vs 21.9 ± 0.2 for LDT (*p* = 0.951), 8.6 ± 4.7 versus 9.2 ± 0.03 for PLTi (*p* = 0.146), 9.8 ± 0.8 vs 10.1 ± 0.04 for PLTd (*p* = 0.600), 19.7 ± 0.13 vs 19.8 ± 0.01 for Barthel (*p* = 0.792) and 13.3 ± 0.31 vs 13.6 ± 0.02 for IADL (*p* = 0.172).

**Table 2:** Cognitive Function at Baseline Over Strata of CKD stages and Overall

	**Total** ***n*** **= 5796**	**CKD stages based on eGFR (ml/min/1.73m**^**2**^**)**				*P*-value*
		**Stage 4** ***n*** **= 19**	**Stage 3b** ***n*** **= 786**	**Stage 3a** ***n*** **= 2306**	**Stage 1 and 2** ***n*** **= 2685**	
Stroop-Colour-Word Test	66.5 ± 0.4	74.2 ± 6.7	66.4 ± 0.9	63.2 ± 0.5	69.3 ± 0.6	< 0.001
Letter-Digit Coding Test	23.1 ± 0.1	21.9 ± 1.2	23.3 ± 0.3	24.4 ± 0.2	21.9 ± 0.2	< 0.001
Picture-Word Learning Test – immediate	9.3 ± 0.03	8.6 ± 4.7	9.3 ± 0.07	9.4 ± 0.04	9.2 ± 0.03	0.001
Picture-Word Learning Test – delayed	10.1 ± 0.04	9.8 ± 0.8	10.0 ± 0.1	10.2 ± 0.05	10.1 ± 0.04	0.197
The Barthel index	19.8 ± 0.01	19.7 ± 0.13	19.7 ± 0.03	19.8 ± 0.01	19.8 ± 0.01	0.004
Instrumental Activities of Daily Living	13.6 ± 0.01	13.3 ± 0.31	13.5 ± 0.04	13.7 ± 0.02	13.6 ± 0.02	0.008

All values are presented as mean ± SE. * *p*-values of differences between groups were assessed using an one-way ANOVA test. Abbreviations: *CKD* Chronic kidney disease, *eGFR* estimated glomerular filtration rate, *SE* Standard error

**Fig. 1:**
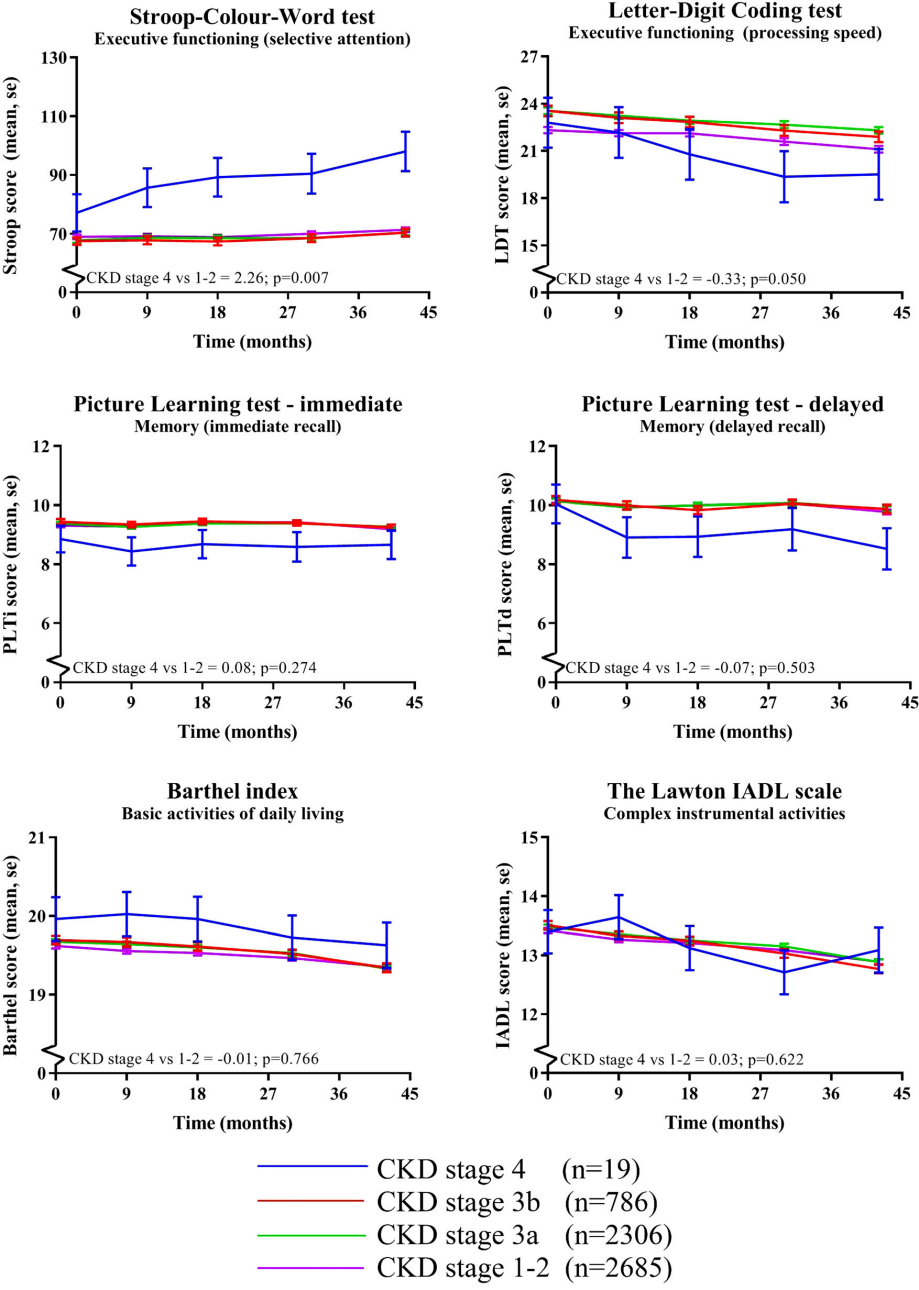
Effect of CKD stage on cognitive function and functional status over time. * Means were assessed using linear mixed models adjusted for prespecified variables including sex, age, educational status, country, statin treatment and multiple other known vascular confounders. *P*-values represent the statistical significance of the difference in cognitive test score changes over time between CKD stage 4 (eGFR < 30 ml/min/1.73m^2^) versus CKD stage 1–2 (eGFR> 60 ml/min/1.73m^2^). Abbreviations: Barthel, the Barthel index; eGFR, estimated glomerular filtration rate; IADL, Instrumental Activities of Daily Living; LDT, Letter-Digit Coding Test; PLTd, Picture-Word Learning Test – delayed; PLTi, Picture-Word Learning Test – immediate; Stroop, Stroop-Colour-Word Test

Mean follow-up was 42 months with a range of 36–48 months. Figure 1 shows the effect of CKD stage on the different cognitive function and functional status tests over time. The mean cognition and functional status scores are adjusted for all prespecified confounders. In all cognitive function tests, a trend was seen for faster cognitive decline over time in CKD stage 4 compared to the other CKD groups. No differences were seen for functional status. When comparing the most severe CKD stage 4 (< 30 ml/min/1.73m^2^) versus stage 1–2 (> 60 ml/min/1.73m^2^) over time the estimates (95% confidence interval (CI)) are 2.26 (0.63–3.88; *p* = 0.007) for Stroop, − 0.33 (− 0.66–0.00; *p* = 0.050) for LDT, 0.08 (− 0.06–0.21; *p* = 0.274) for PLTi, − 0.07 (− 0.27–0.13; *p* = 0.503) for PLTd, − 0.01 (− 0.11–0.08; *p* = 0.766) for Barthel and 0.03 (− 0.09–0.15; 0.622) for IADL, see also Fig. 1. Participants with mild to modest CKD stage 3 compared to CKD stage 1–2 had no worse cognitive function, which is also seen in Fig. 1, displaying practically parallel lines for CKD stages 3 to 1.

### Stratification for history of vascular disease

**Fig. 2:**
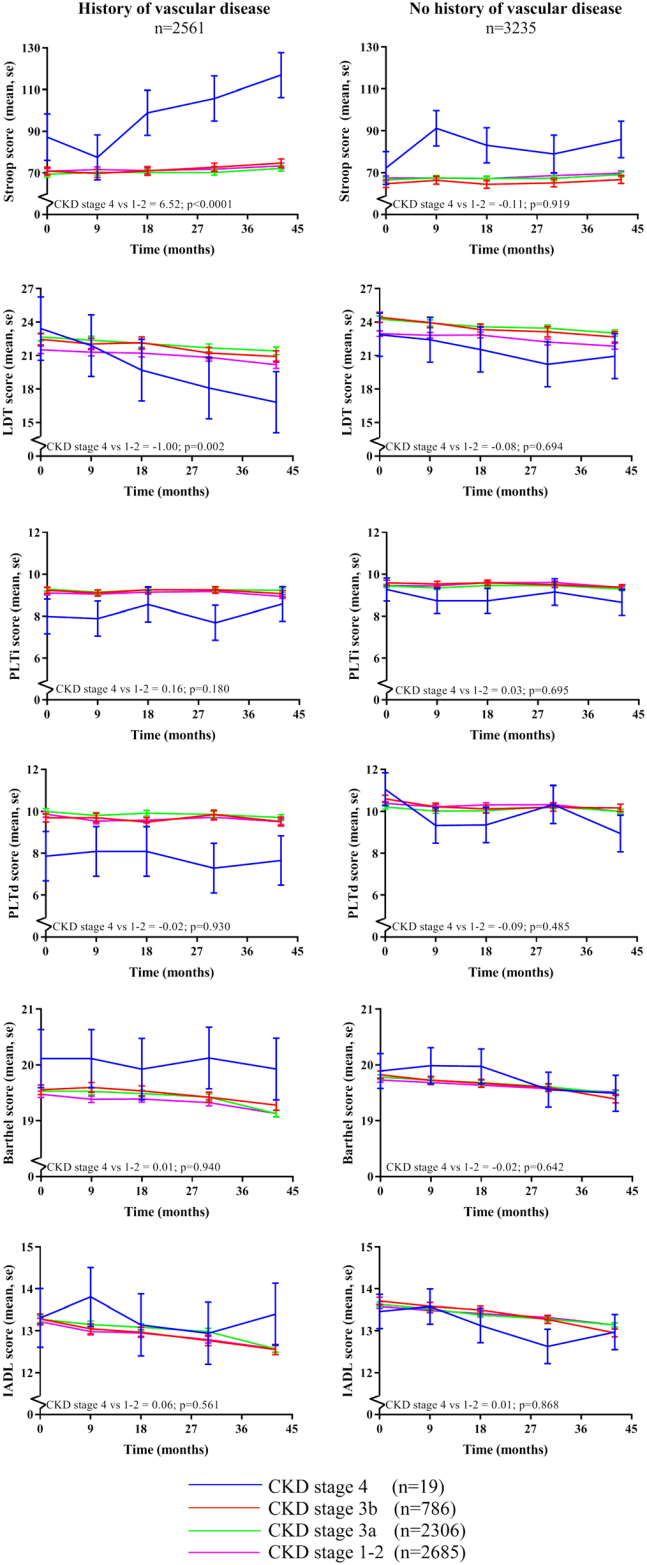
Effect of CKD stage on cognitive function and functional status over time stratified for history of vascular disease. * Means were assessed using linear mixed models adjusted for prespecified variables including sex, age, educational status, country, statin treatment and multiple other known vascular confounders. P-values represent the statistical significance of the difference in cognitive test score changes over time between CKD stage 4 (eGFR < 30 ml/min/1.73m^2^) versus CKD stage 1–2 (eGFR> 60 ml/min/1.73m^2^). Abbreviations: Barthel, the Barthel index; eGFR, estimated glomerular filtration rate; IADL, Instrumental Activities of Daily Living; LDT, Letter-Digit Coding Test; PLTd, Picture-Word Learning Test – delayed; PLTi, Picture-Word Learning Test – immediate; Stroop, Stroop-Colour-Word Test

In Fig. 2 the analysis was stratified according to the history of vascular disease. The trend of faster cognitive decline over time in CKD stage 4 compared to the other CKD groups was most prevalent in patients with a history of vascular disease compared to patients without a history of vascular disease, see Fig. 2 and Table 3. No differences were found for functional status. Estimates (95% CI) of CKD stage 4 versus stage 1–2 in patients with a history of vascular disease are 6.52 (3.94–9.10; *p* < 0.0001) for Stroop, − 1.00 (−1.62 – − 0.37; *p* = 0.002) for LDT, 0.16 (− 0.08–0.40; *p* = 0.180) for PLTi, − 0.02 (− 0.37–0.34; *p* = 0.930) for PLTd, 0.01 (− 0.16–0.18; *p* = 0.940) for Barthel and 0.06 (− 0.15–0.28; *p* = 0.562) for IADL. Estimates (95% CI) of CKD stage 4 versus stage 1–2 in patients without a history of vascular disease are − 0.11 (− 2.21–1.99; *p* = 0.919) for Stroop, − 0.08 (− 0.47–0.32; *p* = 0.694) for LDT, 0.03 (− 0.13–0.20; *p* = 0.695) for PLTi, − 0.09 (− 0.33–0.15; *p* = 0.485) for PLTd, − 0.02 (− 0.13–0.18; *p* = 0.642) for Barthel and 0.01 (− 0.12–0.15; *p* = 0.868) for IADL, see also Table 3. Corresponding *p*-values for interaction of vascular disease and cognitive decline or functional decline over time were 0.016 for Stroop, 0.115 for LDT, 0.529 for PLTi, 0.123 for PLTd, 0.737 for Barthel and 0.064 for IADL.

**Table 3:** Effect of CKD stage 4 versus stage 1–2 on cognitive function over time

	**Total**		**History of vascular disease**		**No history of vascular disease**		**interaction**
	**Beta (95%CI)**	***p*-value**	**Beta (95%CI)**	***p*-value**	**Beta (95%CI)**	***p*-value**	***p*-value**
Stroop-Colour-Word Test	2.26 (0.63–3.88)	0.007	6.52 (3.94–9.10)	< 0.0001	− 0.11 (− 2.21–1.99)	0.919	0.016
Letter-Digit Coding Test	− 0.33(− 0.66–0.00)	0.050	−1.00 (− 1.62 – − 0.37)	0.002	−0.08 (− 0.47–0.32)	0.694	0.115
Picture-Word Learning Test – immediate	0.08 (− 0.06–0.21)	0.274	0.16 (− 0.08–0.40)	0.180	0.03 (−0.13–0.20)	0.695	0.529
Picture-Word Learning Test – delayed	−0.07 (− 0.27–0.13)	0.503	−0.02 (− 0.37–0.34)	0.930	−0.09 (− 0.33–0.15)	0.485	0.123
The Barthel index	−0.01 (− 0.11–0.08)	0.766	0.01 (− 0.16–0.18)	0.940	−0.02 (− 0.13–0.08)	0.642	0.737
Instrumental Activities of Daily Living	0.03 (− 0.09–0.15)	0.622	0.06 (− 0.15–0.28)	0.561	0.01 (− 0.12–0.15)	0.868	0.064

* *p*-values of differences in cognitive test score changes over time were assessed between CKD stage 4 (eGFR < 30 ml/min/1.73m^2^) versus CKD stage 1–2 (eGFR > 60 ml/min/1.73m^2^) using linear mixed models adjusted for prespecified variables including sex, age, educational status, country, statin treatment and multiple other known vascular confounders

## Discussion

In this large cohort of older people with an increased risk for, or a history of, vascular disease, only severe kidney disease (CKD stage 4), but not mild to modest kidney disease (CKD stage 3a and b), was associated with cognitive impairment at baseline and cognitive decline over time. The association of severe kidney disease with cognitive impairment and decline over time was more outspoken in patients with a history of vascular disease. No association was found between kidney function and functional status.

Severe kidney failure as independent risk factor for cognitive dysfunction, in combination with lack of effect of the association of mild to modest kidney failure, has been found in previous research [29, 30]. This might be due to the fact that nephrogenic risk factors only start playing a role in more advanced CKD [13]. Patients that do have cognitive dysfunction in earlier stages of CKD might have worse cognitive function mainly related to vascular damage. Because it is known from previous studies that a history of vascular disease or risk factors as hypertension can lead to microvascular damage and small vessel disease in the brain, CKD can ultimately lead to cognitive dysfunction via this pathway [8, 9]. Furthermore, impaired cardiac function, measured by NT-proBNP or Troponin, is associated with cognitive dysfunction independently from other cardiovascular risk factors [31–33]. As expected, also in this study, a potential predictor for worse cognitive function at baseline in multivariate analysis was a higher vascular burden, as shown before in this cohort [19, 34].

The contribution of kidney failure to cognitive dysfunction on top of a high vascular burden versus no vascular burden remained hitherto mostly unknown. In general, it is difficult to make a distinction between cognitive decline in CKD patients with or without vascular risk factors, because many CKD patients have a vascular aetiology of their kidney failure, for instance due to hypertension or diabetes mellitus. In this cohort 44.2% of participants were included with a history of vascular disease, 61.9% had hypertension and 10.7% had diabetes. Therefore, we used stratification to distinguish patients with proven vascular disease from patients with only vascular risk factors. Cognitive function declined faster over time in patients with CKD stage 4 (< 30 ml/min/1.73m^2^) especially together with a history of vascular disease. In a comparable subanalysis, Seidel et al. also showed increasing prevalence of depression and cognitive dysfunction in the CVD group (defined as coronary heart disease or myocardial infarction) compared to no CVD in the higher CKD-stages compared to controls [35].

As previous research has been shown, decline in kidney function associates with a higher risk of stroke, partly due to a higher incidence in atrial fibrillation [36, 37]. Approximately 10% of patients develop new-onset dementia after first stroke, and even more after recurrent stroke [38, 39]. According to the U.S. Renal Data System prevalence of atrial fibrillation in patients with or without CKD is 24.0% versus 9.5% and prevalence of CVA or TIA is 19.4% versus 7.7%. Prevalence of both diseases increases with increasing stage of kidney failure [40]. Therefore, a history of stroke or TIA, which is one of the inclusion criteria in this study, could be a confounder considering the influence on cognitive impairment. In the total group 11.2% had a history of stroke or TIA, which did not differ significantly between groups (*p* = 0.584); 1 (5.3%), 86 (10.9%), 271 (11.8%) and 289 (10.8%) in the CKD stage 4, stage 3b, stage 3a and stage 1–2 respectively. Therefore, the worse cognitive function in CKD stage 4, appears unlikely to be explained by the prevalence of stroke or TIA.

Established previously in PROSPER, impaired kidney function was independently associated with increased risk of all-cause mortality, fatal vascular events and with composite fatal and nonfatal coronary and heart failure outcomes. This effect was most prevalent in eGFR < 40 ml/min/1.73m^2^. Notably, the PROSPER investigators did not find an association between an impaired eGFR and a higher risk of stroke [41]. However, not only impaired kidney function increases the risk of mortality, but it is also known, that next to the importance to prevent cognitive decline for better quality of life of patients, a worse cognitive function associates with higher morbidity and mortality rates in older patients reaching end-stage kidney disease [42–44].

The difference in cognitive decline for CKD stage 4 and CKD stage 1–2 might be partially explained by the sex difference between the groups. However, a sex-difference in cognitive decline is debated. A review showed that sex did not determine the rate of cognitive decline between ages of 60–80 years, an age that is comparable to our cohort that included patients between 70 and 82 years [45].. In our cohort univariate male sex correlated with worse cognitive function, however multivariate analysis showed still worse memory tests compared to females, but better cognitive function in executive function (data not shown). These results are comparable with a study by Proust-Lima et al., whereby older women showed better outcomes in memory tests, whereas men had a better visuospatial ability [46]. Therefore, the lack of males in the CKD stage 4 cannot explain the worse outcome in that category.

### Limitations

Our study has several limitations. First of all, our population was selected for a specific clinical trial and do not represent the general population.

Another limitation is that we were restricted to eGFR in our measures of kidney function, other predictors as albuminuria or cystatin C are not available. A recent systematic review and meta-analysis of prospective, population-based studies, showed that albuminuria was most consistent as marker of CKD in the association with cognitive decline with an odds ratio of 1.35 (95% CI 1.06–1.73). An eGFR < 60 ml/min/1.73m^2^ showed no significant association with cognitive dysfunction, consistent with our results, with an odds ratio of 1.28 (95% CI 0.99–1.65) [47]. In our cohort the same analyses with creatinine instead of eGFR yielded similar results that were inversely proportional as expected (data not shown). Furthermore, no distinction can be made between acute or chronic kidney failure. Creatinine was only measured at baseline, so no follow-up measurements are available, therefore it is not known whether patients with a normal kidney function at baseline deteriorated or patients with an impaired kidney function improved over time. In addition, it would be interesting to see whether patients with a faster cognitive decline also have a faster decline in kidney function, considering that both the brain and kidney share similar hemodynamic characteristics, since both are low resistance end organs exposed to high-volume blood flow and therefore predisposed for vascular damage [4, 48].

Whereas one of the exclusion criteria was a high creatinine at baseline and the groups of eGFR were divided based on clinical stages, the group containing CKD stage 4 was relatively small (after stratification only seven participants with and 12 participants without a history of vascular disease). Therefore, although the results of CKD stage 4 appear clinically relevant, especially in the cognitive function analysis over time (Figs. 1 and 2), significance remained partly absent and therefore results needs to be interpreted with caution. Furthermore, although the results of the cognitive function tests show the same trend in both cognitive domains, the effect on executive functioning seems statistically larger than the effect on memory. Possible explanation could be that executive functioning seems most often sooner affected than memory, partly due to more sensitive executive function tests, and memory would be more affected over a longer follow-up period.

## Conclusions

In this study, only severe kidney disease (CKD stage 4) seem to be associated with cognitive impairment at baseline and cognitive decline over time. A mild to modest impaired kidney function appeared not to be independently associated with cognitive decline during a 3.2 year follow-up period. For better understanding of the mechanisms involved in cognitive decline in CKD, especially end-stage kidney disease, additional studies are necessary, which may contribute to interventions for prevention. Combined (metabolic) parameters of kidney function, beside eGFR, should be taken into account, as well as albuminuria. An observational study is currently under way to gain insight in the potential different mechanisms of cognitive decline in older patients with end-stage kidney disease to identify modifiable risk factors [49]. Furthermore, it would be of great interest to determine whether newer agents which seem to meaningfully slow renal function decline such as SGLT2 inhibitors, also slow decline in cognitive function, although we accept such agents may also lessens risks in cardiovascular disease [50].

## Data Availability

The datasets used and/or analysed during the current study are available from the corresponding author on reasonable request.

## Abbreviations

Barthel: The Barthel index
BMI: Body mass index
CI: Confidence interval
CKD: Chronic kidney disease
CRP: C-reactive protein
CVA: Cerebrovascular accident
DBP: Diastolic blood pressure
eGFR: estimated glomerular filtration rate
Hb: Hemoglobin
HDL: High-density lipoprotein
IADL: Instrumental Activities of Daily Living
LDL: Low-density lipoprotein
LDT: Letter-Digit Coding Test
MMSE: Mini-Mental State Examination
NT-proBNP: N-terminal pro b-type natriuretic peptide
OR: Odds ratio
PLTd: Picture-Word Learning Test – delayed recall
PLTi: Picture-Word Learning Test – immediate recall
PROSPER: The PROspective Study of Pravastatin in the Elderly at Risk
SBP: Systolic blood pressure
Stroop: Stroop-Colour-Word Test
TIA: Transient ischemic attack
